# Anaemia prevalence and risk factors among nonpregnant and pregnant women of reproductive age in Ghana: an analysis of the Ghana demographic and health survey data

**DOI:** 10.1186/s41182-025-00792-8

**Published:** 2025-08-27

**Authors:** Gangtaba Gilbert Agulu, Noudéhouénou Crédo Adelphe Ahissou, Yasuhiko Kamiya, Frank Baiden, Mitsuaki Matsui

**Affiliations:** 1 School of Tropical Medicine and Global Health, Nagasaki University, 1-12-4 Sakamoto, Nagasaki, 852-8523 Japan; 2 Department of Global Health, Graduate School of Health Sciences, University of the Ryukyus, 1076 Kiyuna, Nishihara, Okinawa 901-2027 Japan; 3 Fred N Binka School of Public Health, University of Health and Allied Science, PMB 31, Hohoe, Volta Region Ghana; 4 Graduate School of Health Science, Kobe University, 7-10-2 Tomogaoka, Suma-Ku, Kobe, Hyogo 654-0142 Japan

**Keywords:** Anaemia, GDHS, Nonpregnant, Pregnant, Women of reproductive age

## Abstract

**Background:**

Despite extensive global and national efforts to reduce anaemia, it remains a major public health concern among Women of Reproductive Age (WRA). However, community-based studies that compare the prevalence and risk factors of anaemia using nationally representative samples are limited in Ghana. This study examines and compares anaemia prevalence and associated risk factors between nonpregnant and pregnant WRA in Ghana.

**Methods:**

This study utilized cross-sectional data from the 2022 Ghana Demographic and Health Survey (GDHS). A total of 15,014 WRA were recruited, of whom 7,557 were screened for anaemia including 7004 nonpregnant and 553 pregnant women. Anaemia was defined as haemoglobin levels below 12 g/dL for nonpregnant and below 11 g/dL for pregnant women. Pearson chi-square and Fisher’s exact tests were used to compare anaemia prevalence across groups. Poisson regressions were applied to identify risk factors for anaemia. All analyses were conducted using Stata version SE.18.

**Results:**

The prevalence of anaemia was 40.4% among nonpregnant women and 51.4% among pregnant women. Aside from self-reported health status and toilet facilities being significant determinants for nonpregnant women, common factors affecting both groups included parity, BMI, wealth status, and geographic zone. Multiparous women had a higher risk of anaemia, with nonpregnant and pregnant women experiencing 23% and 43% increased risk, respectively. Underweight nonpregnant women had an 11% higher risk, while overweight pregnant women had a 34% lower risk of anaemia. In terms of wealth, women in the poorest quintile had a significantly higher risk of anaemia 36% among nonpregnant women (APR: 1.36, 95% CI 1.01–1.83, p = 0.049) and 32% among pregnant women (APR: 1.32, 95% CI 1.01–1.76, p = 0.049). Additionally, women residing in the northern zone had a higher anaemia risk compared to those in the southern zone. Among nonpregnant women, those reporting poor health status had a 51% increased risk of anaemia, while those with improved toilet facilities had a 10% lower risk (APR: 0.90, 95% CI 0.83–0.96, p = 0.004).

**Conclusions:**

The prevalence of anaemia, particularly among pregnant women, remains high in Ghana and constitutes a significant public health threat. Addressing this issue requires holistic and tailored public health strategies that improve access to healthcare, nutrition, sanitation, and economic equity.

**Supplementary Information:**

The online version contains supplementary material available at 10.1186/s41182-025-00792-8.

## Background

Anaemia is a debilitating haemological condition characterized by a deficit in the red blood cells or haemoglobin (Hb) concentration below the normal thresholds [1]. It is often defined based on Hb cut-off values adjusted for gender, age, smoking habits, physiological status, and altitude [2]. Concerning gestational status, the World Health Organization (WHO) defines anaemia as Hb concentration < 12.0 g/dL for nonpregnant women and < 11.0 g/dL for pregnant women [1, 3].

Globally, anaemia remains a major public health concern, particularly among Women of Reproductive Age (WRA), affecting 30% of nonpregnant women and 37% of pregnant women in 2019 [4].

Pregnancy increases iron demands, making pregnant women more vulnerable to anaemia and its complications. While the WHO recommends Iron and Folic Acid (IFA) supplementation for pregnant women to prevent anaemia, adherence to these guidelines remains low, which can lead to adverse maternal and child health outcomes [5]. For instance, only 41% of pregnant women in Ghana and 47% in Ethiopia adhered to IFA supplementation [2, 5]. Barriers to adherence include misconceptions about the benefits of IFA, forgetfulness, side effects such as nausea and constipation, financial constraints, and limited availability of the supplements [5, 6]. Improving adherence to IFA supplementation requires addressing personal and systemic barriers through early ANC attendance, education, side effect management, family support, and improved access [7, 8].

Several factors increase the risk of anaemia in pregnancy, including inadequate nutritional intake—particularly iron deficiency, underlying health conditions such as chronic kidney disease and autoimmune disorders, closely spaced pregnancies, multiple gestations (twins or triplets), a history of heavy menstrual bleeding, and persistent vomiting during pregnancy [2].

Pregnant anaemic women face a greater risk of maternal complications, such as heart failure, premature delivery, and postpartum haemorrhage, with a 3.5 times higher risk of mortality in severe anaemia [9, 10]. Additionally, anaemia contributes to poor perinatal outcomes—foetal growth restriction, stillbirth, neonatal asphyxia, and neonatal death, with incidences of 62% in anaemic women compared to 28% in nonanaemic women [9–12].

Although nonpregnant women do not experience the iron and blood losses associated with pregnancy, childbirth, or the postpartum period, they remain at risk of anaemia due to factors such as regular menstrual blood loss, inadequate dietary iron intake, and physically demanding activities that may increase iron demands or losses [13, 14]. As a result, many women, particularly in developing countries enter pregnancy already anaemic [15, 16].

Anaemia is more prevalent in low- and middle-income countries (LMICs) due to global disparities in nutrition, education, and access to healthcare, which tend to be less favourable in these countries [9, 17]. For example, in sub-Saharan Africa, the current prevalence in WRA is 43%, with a much higher Fig. (51%) in Western Africa [18]. According to the WHO 2021 Global Health Observatory data, high rates were reported in Mali (59%), Benin (55.2%), and Gabon (51.9%), while Ethiopia (23.9%) and Rwanda (17.2%) had comparatively lower rates [19]. Between 2000 and 2019, nearly all African countries, except Burundi, Mauritius, and Tunisia, demonstrated some level of reduction in the prevalence in WRA [19]. In Ghana, data from the Ghana Demographic and Health Survey (GDHS) revealed a marginal decline among WRA over the years, from 45% in 2003 to 41.1% in 2022. Despite this trend, anaemia prevalence among pregnant women in Northern Ghana remains high, with estimates of nearly 60% of pregnant women being affected [20].

Reducing anaemia prevalence among WRA has been a longstanding target for Ghana. To achieve global and national anaemia reduction targets, concerted efforts have been made by the Ministry of Health and other agencies to implement interventions, including universal daily IFA supplementation, deworming, and malaria prevention programmes. Additionally, in 2018, the Ghana government enacted the Girls’ Iron-Folate Tablet Supplementation (GIFTS) programme to control anaemia among adolescent girls [21]. Notwithstanding these efforts, anaemia is still a pressing public health issue in Ghana, affecting women from poor and marginalized communities.

To date, most anaemia studies have focused separately on nonpregnant and pregnant women. To the best of our knowledge, no comparative study has examined the prevalence and determinants of anaemia among both groups using nationally representative data from Ghana. Therefore, this study is crucial for identifying and comparing the prevalence and risk factors of anaemia among nonpregnant and pregnant women in Ghana, using data from the 2022 GDHS. Understanding these predictors among WRA in Ghana is essential for designing targeted interventions to reduce anaemia prevalence and improve maternal health outcomes.

## Methodology

### Study site

**Fig. 1:**
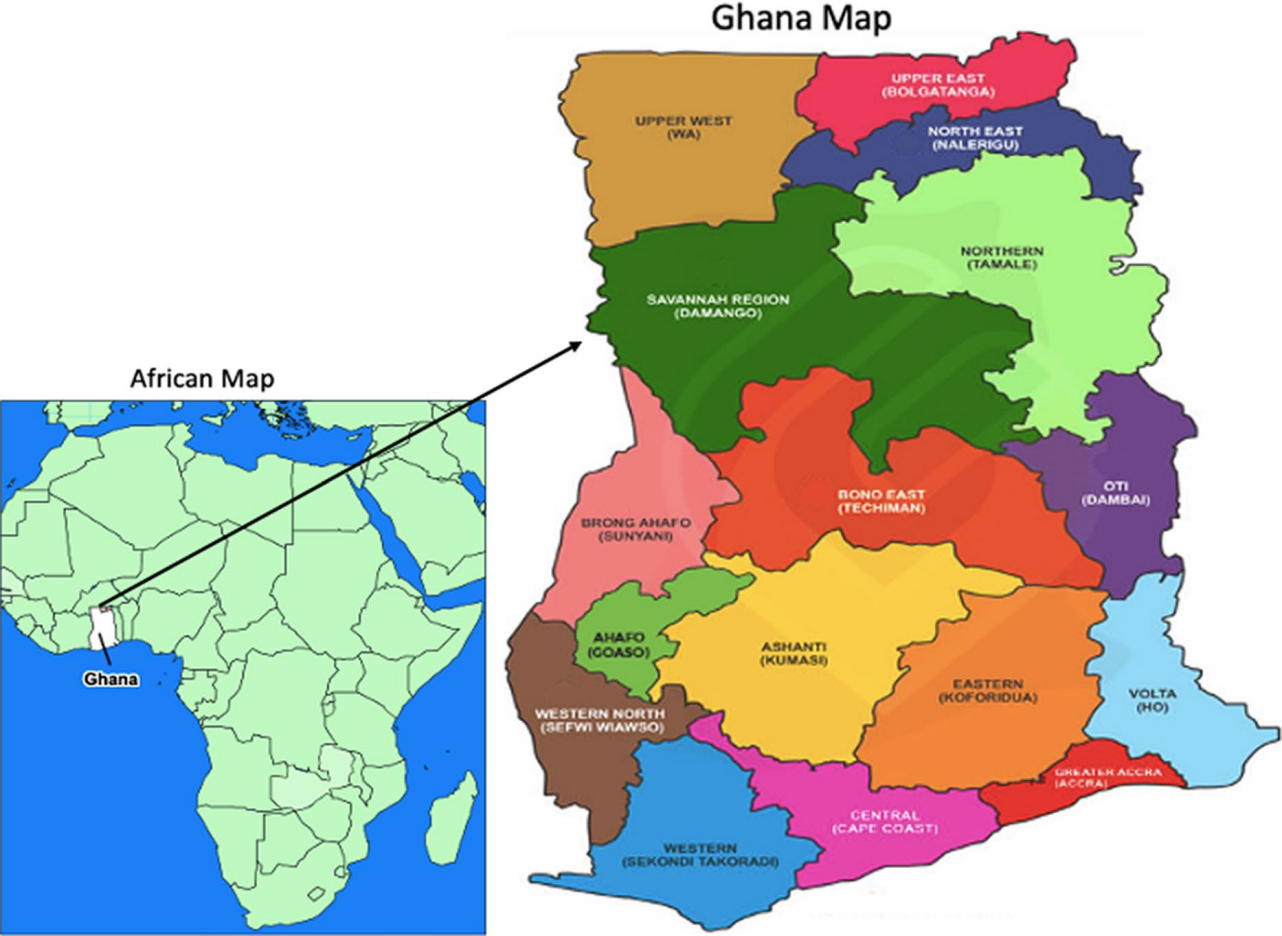
Map of the study area, adapted from OpenStreetMap, with modification

Ghana is situated in West Africa, along the shores of the Gulf of Guinea (Fig. 1). It is geographically categorized into three main zones, comprising 16 regions: Southern (Western, Central, Greater Accra, Volta, and Eastern), Middle (Ashanti, Western–North, Ahafo, Bono, Bono–East, and Oti), and Northern (Northern, Savanna, North–East, Upper East, and Upper West). The 2021 Population and Housing Census revealed an estimated national population of 30.8 million, with a 6.1 million increase since 2010. The country has 261 administrative districts with about 60% of the population living in urban areas [22]. Healthcare availability in Ghana varies significantly across regions, with rural areas often facing challenges such as fewer healthcare facilities, limited resources, and longer distances to healthcare centres compared to urban areas [23]. Approximately 68.6% of Ghana's population is covered by either the National Health Insurance Scheme (NHIS) or private health insurance schemes [24, 25]. Ghana currently has an estimated 7,152,901WRA, with a ratio of five midwives per 1,000 live births. Maternal mortality accounts for 14% of all deaths, with 10% of these deaths attributed to direct maternal causes [26].

Ghana was chosen as the study site because anaemia affects over 40% of WRA, surpassing the WHO threshold for classification as a severe public health issue [27]. This condition has implications for maternal and child health, yet Ghana's progress in addressing it remains limited despite several ongoing interventions. Additionally, Ghana benefits from high-quality, nationally representative data from the Demographic and Health Surveys (DHS), making it an ideal setting for examining anaemia prevalence and risk factors. Ghana’s varied geographic (urban and rural) and sociodemographic contexts offer a suitable setting for exploring factors that influence anaemia risk, with findings likely to be relevant to other LMICs [27].

### Study design and data source

This study used cross-sectional data from the 2022 GDHS collected from 17 October 2022 to 14 January 2023. The GDHS is conducted by the Ghana Statistical Service (GSS) and the Ghana Health Service (GHS), with technical assistance from ICF (formerly ICF International), and is funded by the United States Agency for International Development (USAID) and the U.S. President’s Malaria Initiative (PMI).

The 2022 survey contributes to the seventh round of the DHS series conducted in Ghana to date, with previous rounds occurring approximately every five years: 1988, 1993, 1998, 2003, 2008, and 2014 [27]. Four sets of questionnaires were designed for this survey, including women, men, household, and biomarkers questionnaires [27]. We extracted the dataset in March 2024 for this study.

### Sampling strategy

The 2022 GDHS employed a stratified two-stage cluster sampling design across the 16 regions of the country. In the first stage, 618 clusters were selected using probability proportional to size (PPS) sampling covering both rural and urban areas. In the second stage, households within the selected clusters were mapped and listed to create a sampling frame for selection. A total of 18,450 households were selected from which 15,014 women aged 15–49 were successfully interviewed, yielding a response rate of 98% [27].

### Study population and sample size

The study population included nonpregnant and pregnant WRA. Of the 15,014 WRA recruited, 7557 were screened for anaemia comprising 7004 nonpregnant and 553 pregnant women.

### Anthropometric measurement and blood testing

The biomarkers assessed in the 2022 GDHS included Body Mass Index (BMI), anaemia, and malaria. Anthropometric measurements—weight and height were taken using a SECA 874U scale and a ShorrBoard^®^ measuring board, respectively. For anaemia screening, blood samples were obtained via finger prick and collected into micro cuvettes. Haemoglobin levels were analysed on-site using a battery-operated HemoCue^®^ 201 + device and results were immediately communicated to respondents [27]. Individuals diagnosed with severe anaemia were referred to a health facility for further assessment and treatment. Notably, malaria testing was conducted only among children aged 6–59 months [27].

### Variables

#### Outcome variable

The primary outcome variable for this study was anaemia status, which was determined by Hb levels among nonpregnant and pregnant women. For nonpregnant women, it was classified as severe (< 8.0 g/dL), moderate (8.0–10.9 g/dL), mild (11.0–11.9 g/dL), or no anaemia (≥ 12.0 g/dL). For pregnant women, anaemia was classified as severe (< 7.0 g/dL), moderate (7.0–8.9 g/dL), mild (9.0–10.9 g/dL), or no anaemia (≥ 11.0 g/dL) [28]. Anaemia status was further dichotomized using WHO thresholds based on Hb concentrations into ‘anaemic’ and ‘nonanaemic’ to facilitate analysis using a binary Poisson regression model. Women were classified as either ‘anaemic’ (Hb < 12.0 g/dL for nonpregnant women; < 11.0 g/dL for pregnant women) or ‘nonanaemic’ (Hb ≥ 12.0 g/dL for nonpregnant women; ≥ 11.0 g/dL for pregnant women) in the multivariate Poisson regression model [28].

Haemoglobin measurements were adjusted for altitude and cigarette smoking in study areas located over 1,000 m above sea level [27, 29]. The adjustment was calculated using the formula:

 adjust = –0.032 × alt + 0.022 × alt, and. adjusted Hb (adjHb) = Hb – adjust (when adjust > 0),

where Hb is measured in grammes per decilitre (g/dL) and altitude (alt) is in thousands of metres above sea level.

### Exposure variables

Exposure variables were selected based on a review of the GDHS questionnaires and relevant literature [17, 30, 31]. The primary independent variable was pregnancy status, which was treated as a binary variable. All other explanatory variables were stratified by pregnancy status. A full list of the variables is provided in the supplementary materials file (Supplementary Table S1).

### Data processing and analysis

Before data analysis, the dataset was cleaned and checked for inconsistencies and missing values. Sampling weights were applied to all cases using the ‘svyset’ command to ensure that the results were representative of the population [32]. Data analyses were conducted using Stata version SE 18.0. Descriptive statistics were computed for sociodemographic variables and are presented as frequencies and percentages. Pearson’s chi-square test was used to examine associations between categorical variables and anaemia prevalence [33]. Where expected cell counts were less than five, Fisher’s exact test was used. Independent variables were assessed for multicollinearity using the Variance Inflation Factor (VIF), and no significant collinearity was detected, with a mean VIF of 2.0 (Supplementary Table S2).

Only variables with a p-value ≤ 0.05 in the bivariable logistic regression analysis were included in the multivariable analysis. A Poisson regression model with robust standard errors was employed to estimate Prevalence Ratios (PRs) for anaemia, as this method is recommended for cross-sectional studies with common binary outcomes—particularly when the outcome prevalence exceeds 10%. Results from the Poisson regression were reported as Crude Prevalence Ratios (CPR) and Adjusted Prevalence Ratios (APR), each with the corresponding 95% Confidence Intervals (CIs). The Likelihood Ratio Test (LRT) was used to determine the best-fitting model.

### Missing data

Missing data were identified and excluded from the analysis. Codes like ‘inconsistent’, ‘missing’, ‘don’t know’, and ‘not applicable’ were left out when computing statistics such as means or medians. The sample size varies across some variables in the results section (minimum dietary diversity, toilet facility, source of drinking water, geographic zone, and partner occupation) due to the exclusion of missing values. However, this did not cause major distortions in the estimation of anaemia prevalence; hence, the results remain valid.

### Ethical approval

The 2022 GDHS received ethical approval from the Ghana National Health Research Ethics Committee (NHREC) and the ICF Institutional Review Board. Informed consent was obtained from all participants before interviews and biomarker testing [27]. As this study is based on secondary data analysis, no additional ethical approval was required.

## Results

### Background characteristics of respondents

This study analysed the prevalence and predictors of anaemia among nonpregnant and pregnant WRA in Ghana, considering their sociodemographic characteristics (Table 1). The analysis was based on a weighted sample of 15,014 WRA, comprising 13,903 nonpregnant and 1,111 pregnant women, extracted from the individual recode dataset of the 2022 GDHS.

**Table 1:** Sociodemographic characteristics of WRA by pregnancy status (N = 15,014) (2022 GDHS)

Variables	Nonpregnant women N = 13,903		Pregnant women N = 1,111	
	n	%	n	%
Individual level variables				
Age (years)				
15–19	2,756	18.8	79	4.9
20–24	2,403	17.7	266	21.9
25–29	2,089	14.6	297	29.1
30–34	1,979	14.4	249	23.8
35–39	1,868	13.6	156	15.1
40–44	1,593	11.6	53	4.1
45–49	1,215	9.3	14	1.1
Education				
No education	3,078	15.9	279	18.6
Primary	2,052	13.7	193	14.5
Secondary	7,551	60.1	560	58.2
Higher	1,222	10.3	79	8.7
Marital status				
Not married	6,090	47.8	113	11.8
Married	7,813	52.2	998	88.2
Previous experience of giving birth				
No experience	4,417	33.2	241	20.0
Within 24 months before the survey	3,811	24.6	689	17.9
24 months or more before the survey	5,675	42.2	181	62.1
Type of employment				
Unemployed	3,139	22.3	208	15.4
Government employee	2,987	22.3	245	24.4
Self-employed	7,777	55.4	658	60.2
Religion				
Christian	9,712	77.1	718	73.0
Islamic	3,644	19.1	350	23.6
Traditionalist	275	1.8	30	2.6
Others	272	2.0	13	0.8
Number of living children				
0	4,481	33.7	251	20.5
1	2,198	16.0	261	26.0
2–4	4,966	35.9	488	44.8
5 +	2,258	14.4	111	8.7
Self-reported health status				
Very bad	4,551	31.4	328	28.7
Bad	6,356	45.3	578	51.4
Moderate	2,470	19.9	178	17.7
Good	456	3.1	24	2.0
Very good	70	0.4	3	0.2
Literacy rate				
Cannot read	6,322	38.9	573	42.7
Can read	7,581	61.1	538	57.3
Body mass index (kg/m^2^)				
Underweight (< 18.5)	572	3.8	14	1.2
Normal (18.5–24.9)	3,880	25.5	307	23.8
Overweight (25.0–29.9)	1,586	12.7	156	14.3
Obese (30 +)	7,865	58.0	634	60.7
ITN usage				
No	7,595	61.1	516	52.4
Yes	6,308	38.9	595	47.6
NHIS coverage				
No	1,310	10.3	45	3.3
Yes	12,593	89.7	1,066	96.7
Alcohol consumption				
No	12,086	85.9	1,028	93.8
Yes	1,817	14.1	83	6.2

Minimum dietary diversity (N = 14,982)	N = 13,875		N = 1,107	
No (not diversified)	7,105	50.2	543	48.4
Yes (diversified)	6,770	49.8	564	51.6

Household/Community level variables				
Zone (N = 14,727)	N = 13,633		N = 1,094	
Southern	5,764	48.9	344	46.7
Middle	3,292	32.6	310	29.5
Northern	4,577	18.5	440	23.8
Residence				
Urban	7,005	42.6	647	48.6
Rural	6,898	57.4	464	51.4
Household head				
Female	5,288	43.4	283	28.9
Male	8,615	56.6	828	71.1
Wealth quintile				
Poorest	3,339	16.1	327	19.7
Poorer	3,120	18.1	246	18.2
Middle	2,802	20.9	206	18.8
Richer	2,491	22.3	195	24.5
Richest	2,151	22.6	137	18.8

Partner occupation status(N = 8,811)	N = 7,813		N = 998	
Not employed	327	4.0	30	2.4
Employed	7,486	96.0	968	97.6

Source of drinking water (N = 14,727)	N = 13,633		N = 1,094	
Unimproved source	5,601	50.2	497	53.8
Improved source	8,032	49.8	597	46.2

Type of toilet facility(N = 14,727)	N = 13,633		N = 1,094	
Unimproved	6,046	33.0	541	35.9
Improved	7,587	67.0	553	64.1

The mean age of both nonpregnant and pregnant women was 29.5 years (SD ± 9.6). More than half of the nonpregnant (7,551; 60.1%) and pregnant (560; 58.2%) women had attained secondary education. The majority in both groups were married (nonpregnant: 7,813; 52.2%, pregnant: 998; 88.2%), self-employed (nonpregnant: 7777; 55.4%, pregnant: 658; 60.2%), Christian (nonpregnant: 9712; 77.1%, pregnant: 718; 73.0%), multiparous (nonpregnant: 4966; 35.9%, pregnant: 488; 44.8%), and literate (nonpregnant: 7581; 61.1%, pregnant: 538; 57.3%).

Most nonpregnant (7595; 61.1%) and pregnant (516; 52.4%) women reported not using Insecticide-Treated Nets (ITNs). A large majority were covered by the Ghana NHIS: 12,593 (89.7%) of nonpregnant and 1066 (96.7%) of pregnant women. Nearly half of the nonpregnant (5764; 48.9%) and pregnant (344; 46.7%) women resided in the southern zone of the country, while 6898 (57.4%) of nonpregnant and 464 (51.4%) of pregnant women lived in rural areas.

Regarding household wealth, 2151 (22.6%) of nonpregnant and 195 (24.5%) of pregnant women belonged to the richer or richest wealth quintiles. In terms of Water, Sanitation, and Hygiene (WASH), slightly more than half of the nonpregnant (5601; 50.2%) and pregnant (497; 53.8%) women relied on unimproved water sources. In contrast, access to improved toilet facilities was reported by 7587 (67.0%) nonpregnant and 553 (64.1%) pregnant women.

### Prevalence and severity of anaemia among nonpregnant and pregnant women (15–49 years) in Ghana (2022 GDHS)

The prevalence and severity of anaemia among both nonpregnant and pregnant women are presented in Tables 2 and 3. Among nonpregnant women, the overall prevalence was 40.4%, with 22.2% classified as having mild anaemia and 1.2% as having severe anaemia. Among pregnant women, the overall prevalence was higher at 51.4%, with 28.6% classified as mild and 22.6% as moderate anaemia.

**Table 2:** Prevalence and severity of anaemia among nonpregnant women (15–49 years) in Ghana (2022–GDHS)

		Anaemia status by haemoglobin level				
Variables	N = 7,004	No anaemia (≥ 12.0 g/dl^3^)	Mild (11.0–11.9 g/dl)	Moderate (8.0–10.9 g/dl)	Severe (< 8.0 g/dl)	
	n(^4^Col.%)	^5^Row %	Row %	Row %	Row %	
Prevalence (40.4%)		59.6	22.2	17.0	1.2	p-value
Individual level variables						
Age (years)						0.005
15–19	1,387(18.7)	56.6	22.2	19.6	1.6	
20–24	1,198(17.4)	63.0	20.7	16.0	0.3	
25–29	1,044(15.0)	62.3	22.5	15.0	0.2	
30–34	1,010(14.3)	62.6	22.5	13.8	1.1	
35–39	930(13.6)	60.2	21.8	16.5	1.5	
40–44	819(11.9)	53.9	23.6	20.3	2.2	
45–49	616(9.2)	56.9	22.9	18.0	2.2	
Education						0.218
No education	1,541(15.8)	56.4	21.6	20.4	1.6	
Primary	1,048(13.9)	58.0	22.0	19.0	1.0	
Secondary	3,818(60.4)	60.5	22.4	15.9	1.2	
Higher	597(9.9)	62.2	21.9	15.0	0.9	
Marital status						0.443
Not married	3,024(47.3)	59.2	21.9	17.4	1.5	
Married	3,980(52.7)	60.0	22.5	16.5	1.0	
Previous experience of giving birth						0.157
No experience	2,197(32.9)	59.4	21.6	17.7	1.3	
Within 24 months before the survey	1,988(25.1)	60.6	23.0	16.1	0.4	
24 months or more before the survey	2,819(42.0)	59.3	22.2	16.9	1.6	
Employment						0.097
Unemployed	1,580(22.4)	58.6	21.0	19.0	1.4	
Government employee	1,467(21.9)	60.0	23.3	14.9	1.8	
Self-employed	3,957(55.7)	59.8	22.3	17.0	0.9	
Religion						0.104
Christian	4,923(77.7)	60.1	21.9	16.6	1.4	
Islamic	1,818(18.8)	57.3	24.3	17.7	0.7	
Traditionalist	123(1.6)	53.7	21.0	24.3	1.0	
Others	140(1.9)	66.5	15.2	18.3	0	
Number of living children						0.082
0	2,231(33.3)	59.3	21.6	17.8	1.3	
1	1,123(16.4)	61.1	23.5	14.6	0.8	
2–4	2,516(36.2)	61.5	21.5	15.7	1.3	
5 +	1,134(14.1)	54.1	23.6	21.0	1.3	
Self-reported health status						< 0.001
Very bad	2,269(31.2)	62.0	22.2	15.0	0.8	
Bad	3,235(46.1)	61.4	21.7	15.9	1.0	
Moderate	1,251(19.2)	53.4	23.5	21.0	2.1	
Good	214(2.9)	53.1	21.7	22.8	2.4	
Very good	35(0.6)	20.8	23.9	47.5	7.9	
Literacy rate						0.329
Cannot read	3,160(38.2)	58.0	22.7	18.0	1.3	
Can read	3,844(61.8)	60.6	21.9	16.3	1.2	
Body mass index (kg/m^2^)						< 0.001
Underweight (< 18.5)	563(7.3)	51.4	22.8	24.6	1.2	
Normal (18.5–24.9)	3,846(49.5)	56.2	23.1	19.3	1.4	
Overweight (25–29.9)	1,576(24.6)	63.5	20.7	14.6	1.2	
Obese (30 +)	1,019(18.6)	66.5	21.6	11.0	0.9	
ITN usage						0.300
No	3,769(60.3)	60.6	22.1	16.2	1.1	
Yes	3,235(39.7)	58.2	22.3	18.2	1.3	
NHIS coverage						0.714
No	649(10.2)	57.5	23.7	17.9	0.9	
Yes	6,355(89.8)	59.9	22.0	16.9	1.3	
Alcohol consumption						0.741
No	6,090(85.8)	59.5	22.5	16.8	1.2	
Yes	914(14.2)	60.6	20.4	17.6	1.4	
Minimum dietary diversity	N = 6,986					0.053
No (not diversified)	3,593(49.6)	59.8	20.9	17.8	1.5	
Yes (diversified)	3,393(50.4)	59.6	23.4	16.0	1.0	
Household/Community level variables						
Zone	N = 6869					0.004
Southern	2,929(49.7)	60.4	21.9	16.1	1.5	
Middle	1,613(32.0)	62.0	20.6	16.7	0.7	
Northern	2,327(18.3)	55.2	23.9	20.4	0.5	
Residence						0.002
Urban	3,523(42.4)	57.8	22.4	19.0	0.8	
Rural	3,481(57.7)	61.0	22.0	15.4	1.6	
Household head						0.593
Female	2,772(45.3)	60.3	21.8	16.5	1.4	
Male	4,232(54.7)	59.0	22.5	17.4	1.1	
Wealth quintile						0.389
Poorest	1,678(15.8)	55.6	22.5	21.0	0.9	
Poorer	1,533(17.8)	59.4	22.5	17.0	1.1	
Middle	1,417(20.5)	59.7	21.3	17.7	1.3	
Richer	1,300(23.3)	61.6	21.4	15.6	1.4	
Richest	1,076(22.6)	60.5	23.5	14.6	1.4	
Partner occupation status	N = 3,980					0.186
Not employed	170(3.9)	59.0	17.1	22.8	1.1	
Employed	3,810(96.1)	60.0	22.7	16.3	1.0	
Source of drinking water	N = 6,869					0.224
Unimproved source	2,853(51.2)	60.0	22.4	16.1	1.5	
Improved source	4,016(48.8)	59.3	21.9	17.9	0.9	
Type of toilet facility	N = 6,869					0.063
Unimproved	3,152(34.0)	56.7	23.4	18.5	1.4	
Improved	3,717(66.0)	61.2	21.5	16.1	1.2	

^*3*^* g/dl* = *Grammes per decilitre, *^*4*^*Col.%* = *Column percentage, *^*5*^*Row%* = *Row percentage*

**Table 3:** Prevalence and severity of anaemia among pregnant women (15–49 years) in Ghana (GDHS 2022)

		Anaemia status by Haemoglobin level				
Variables	N = 553	No anaemia (≥ 11.0 g/dl)	Mild (10.0–10.9 g/dl)	Moderate (7.0–9.9 g/dl)	Severe (< 7.0 g/dl)	
	n**(**Col.%**)**	Row %	Row %	Row %	Row %	
Prevalence (51.4%)		48.6	28.6	22.6	0.2	p-value f*
Individual level variables						
Age(years)						0.298
15–19	37(5.2)	33.1	42.9	24.0	0	
20–24	130(21.1)	43.8	28.3	27.9	0	
25–29	150(28.1)	42.9	37.5	18.9	0.7	
30–34	127(26.0)	56.5	23.0	20.5	0	
35–39	86(16.0)	60.8	19.6	19.6	0	
40–44	18(2.8)	46.5	22.1	31.4	0	
45–49	5(0.8)	10.5	51.2	38.3	0	
Education						0.004
No education	135(17.3)	31.0	30.9	38.1	0	
Primary	96(14.8)	48.8	33.0	18.2	0	
Secondary	285(59.1)	49.2	29.2	21.2	0.4	
Higher	37(8.8)	78.0	12.8	8.5	0	
Marital status						0.898
Not married	48(10.0)	52.7	28.8	18.5	0	
Married	505(90.0)	48.1	28.6	23.1	0.2	
Previous experience of giving birth						0.429
No experience	114(20.6)	44.7	29.5	24.8	1.0	
Within 24 months before the survey	87(16.7)	57.5	27.9	14.6	0	
24 months or more before the survey	352(62.7)	47.5	28.5	24.0	0	
Employment						0.508
Unemployed	97(14.2)	39.7	33.5	26.8	0	
Government employee	123(24.4)	49.4	33.7	16.9	0	
Self-employed	333(61.4)	50.3	25.5	23.9	0.3	
Religion						0.047
Christian	341(71.8)	54.0	27.1	18.9	0	
Islamic	184(23.7)	36.2	31.8	31.1	0.9	
Traditionalist	19(3.2)	29.3	32.7	38.0	0	
Others	9(1.3)	19.0	45.5	35.5	0	
Number of living children						0.461
0	119(21.1)	44.1	30.6	24.4	1.0	
1	142(26.8)	55.6	25.5	18.9	0	
2–4	237(44.0)	49.6	28.2	22.2	0	
5 +	55(8.1)	31.5	36.1	32.4	0	
Self-reported health status						0.319
Very bad	161(29.9)	53.3	27.8	18.9	0	
Bad	276(47.5)	51.1	29.1	19.4	0.4	
Moderate	98(19.3)	37.2	28.1	34.7	0	
Good	15(2.8)	39.1	23.7	37.2	0	
Very good	3(0.5)	21.1	78.9	0	0	
Literacy rate						0.213
Cannot read	295(45.0)	44.4	28.9	26.8	0	
Can read	258 (55.0)	52.0	28.4	19.2	0.4	
Body mass index (kg/m^2^)						0.004
Underweight (< 18.5)	14(2.6)	24.0	57.8	18.2	0	
Normal (18.5–24.9)	305(46.9)	42.2	26.1	31.3	0.4	
Overweight (25–29.9)	153(27.9)	48.1	35.3	16.6	0	
Obese (30 +)	81(22.6)	65.2	22.2	12.6	0	
ITN usage						0.061
No	253(52.1)	54.2	27.2	18.2	0.4	
Yes	300(47.9)	42.5	30.2	27.3	0	
NHIS coverage						0.530
No	20(3.0)	65.4	24.0	10.6	0	
Yes	533(97.0)	48.0	28.8	23.0	0.2	
Alcohol consumption						0.790
No	508(93.5)	48.8	28.9%	22.1%	0.2%	
Yes	45(6.5)	46.3	24.1%	29.6%	0%	
Minimum dietary diversity	N = 1,107					0.70
No (not diversified)	268(46.6)	46.9	29.0	23.6	0.5	
Yes (diversified)	283(53.4)	50.0	28.5	21.5	0	
Household/Community level variables						
Zone	N = 544					< 0.001
Southern	131(45.2)	51.2	30.1	18.7	0	
Middle	197(31.5)	57.3	25.9	16.8	0	
Northern	216 (23.3)	33.4	31.3	34.4	0.9	
Residence						0.023
Urban	326(49.4)	42.6	29.2	28.2	0	
Rural	227(50.6)	54.3	28.1	17.2	0.4	
Household head						0.023
Female	154(31.4)	59.0	22.2	18.1	0.7	
Male	399(68.6)	43.8	31.6	24.6	0	
Wealth quintile						< 0.001
Poorest	161(19.0)	27.8	32.8	39.4	0	
Poorer	128(18.3)	42.3	30.7	25.9	1.1	
Middle	102(19.0)	51.6	28.8	19.6	0	
Richer	91(24.4)	49.5	29.2	21.3	0	
Richest	71(19.3)	70.9	21.7	7.4	0	
Partner occupation status	N = 505					0.86
Not employed	17(2.8)	45.8	37.8	16.4	0	
Employed	488(97.2)	48.3	28.3	23.2	0.2	
Source of drinking water	N = 554					0.236
Unimproved source	247(54.5)	52.7	28.5	18.8	0	
Improved source	297(45.5)	44.5	29.7	25.3	0.5	
Type of toilet facility	N = 554					0.002
Unimproved	268(36.6)	35.8	33.7	30.5	0	
Improved	276(63.4)	56.6	26.4	16.7	0.3	

f* = Fisher’s exact test was conducted where cell count was less than 5

The sociodemographic factors associated with anaemia varied between the two groups; however, common significant variables included BMI, geographic zone, and place of residence. Among nonpregnant women **(**Table 2**)**, significant factors associated with anaemia included age (p = 0.005), self-reported health status (p < 0.001), BMI (p = 0.001), geographic zone (p < 0.001), and residence (p = 0.002). Among pregnant women **(**Table 3**)**, significant factors were education level (p = 0.004), religion (p = 0.047), BMI (p = 0.004), geographic zone (p < 0.001), residence (p = 0.023), type of toilet facility (p = 0.023), household headship (p = 0.0009), and wealth quintile (p = 0.0002).

### Prevalence of anaemia among age groups of WRA in Ghana (2022 GDHS)

**Fig. 2:**
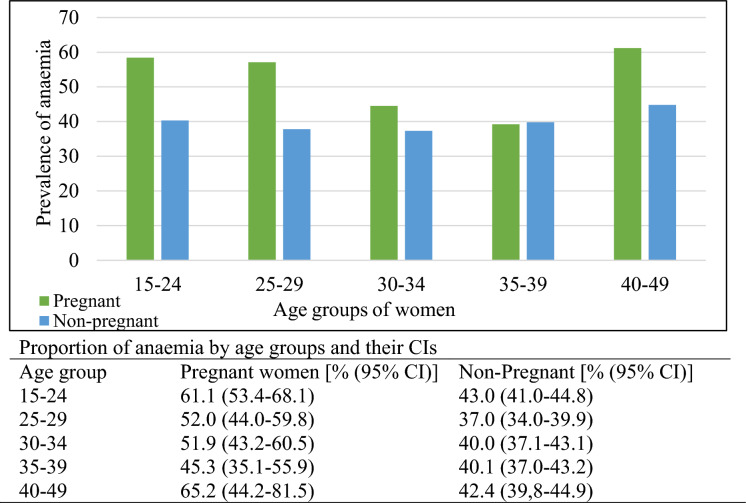
Prevalence of anaemia across different age groups of WRA with 95% CIs

Anaemia prevalence among pregnant women was relatively high across all age groups, peaking at 61.2% in the 40–49 age group and lowest at 39.2% in the 35–39 age group. Among nonpregnant women, the highest prevalence was 44.8% in the 40–49 age group, while the lowest was 37.3% in the 30–34 age group. Overall, anaemia prevalence tended to be higher among the younger and older age groups compared to the middle-aged group of WRA. The confidence intervals (CIs) between age groups indicated no statistically significant differences in anaemia prevalence among pregnant women. However, among nonpregnant women, a significant difference in anaemia prevalence was observed between the 15–24 and 25–29 age groups **(**Fig. 2**)**.

### Multivariate (Poisson regression) results of determinants of anaemia among nonpregnant and pregnant women (15–49 years) in Ghana (2022 GDHS).

Tables 4 and 5 present the multivariable regression results for the determinants of anaemia. Differences in associated risk factors were observed between nonpregnant and pregnant women. After adjusting for potential confounders, number of living children (parity), BMI, geographic zone, and wealth quintile emerged as common predictors significantly associated with anaemia in both groups. Self-reported health status and type of toilet facility were the only variables significantly associated with anaemia among nonpregnant women.

**Table 4:** Multivariate regression results of determinants of anaemia among nonpregnant women (15–49 years) in Ghana (GDHS 2022)

Background variables	Nonpregnant women (N = 7,004)					
	Anaemia		Prevalence ratio (95% CI)			
	Number	prevalence n (%)	CPR^6^ (95% CI)	p- value	APR^7^ (95% CI)	p- value
Total	7,004	2,881 (40.4)	–	–	–	–
Individual level variables						
Age						
15–19	1,387	628(43.4)	1.09(0.96–1.23)	0.180	0.98(0.85–1.13)	0.8563
20–24	1,198	481(37.1)	0.93(0.81–1.06)	0.293	0.92(0.81–1.05)	0.251
25–29	1,044	386(37.8)	0.94(0.82–1.08)	0.451	0.89(0.79–1.01)	0.083
30–34	1,010	405(37.3)	0.93(0.80–1.09)	0.426	0.99(0.89–1.11)	0.957
35–39(Ref^8^.)	930	373(39.8)				
40 +	1,435	608(44.8)	1.12(0.99–1.27)	0.056	1.04(0.95–1.15)	0.338
Education						
No education	1,541	669(43.6)	1.15(1.00–1.31)	0.043	1.00(0.87–1.15)	0.975
Primary	1,048	465(41.9)	1.10(0.95–1.28)	0.176	1.07(0.93–1.23)	0.302
Secondary	3,818	1515(39.6)	1.04(0.92–1.18)	0.475	0.97(0.86–1.10)	0.741
Higher (Ref.)	597	232(37.8)				
Number of living children						
0	2,231	959(40.7)	0.98(0.84–0.88)	0.484	0.78(0.65–0.94)	0.045
1(Ref.)	1,123	448(39.0)				
2–4	2,516	975(38.6)	1.04(0.48–0.92)	0.843	1.02(0.92–1.12)	0.012
5 +	1,134	499(45.9)	1.17(1.04–1.32)	0.006	1.23(0.98–1.21)	< 0.001
Self-reported health status						
Very bad	2,269	914(38.0)	0.81(0.73–0.90)	< 0.001	1.51(1.20–1.90)	< 0.001
Bad	3,235	1284(38.5)	0.82(0.75–0.91)	< 0.001	1.05(0.90–1.22)	0.488
Moderate (Ref.)	1,251	553(46.6)				
Good	214	105(46.8)	1.00(0.81–1.24)	0.967	0.89(0.83–0.96)	0.005
Very good	35	62(79.3)	1.69(1.41–2.03)	< 0.001	0.90(0.83–0.98)	0.022
Body mass index (kg/m^2^)						
Underweight (< 18.5)	563	283(48.7)	1.11(0.99–1.24)	0.058	1.11(1.01–1.22)	0.020
Normal (18.5–24.9) (Ref.)	3,846	1692(43.7)				
Overweight (25–29.9)	1,576	573(36.5)	0.83(0.75–0.92)	< 0.001	0.81(0.75–0.88)	< 0.001
Obese (30 +)	1,019	333(33.6)	0.76(0.68–0.86)	< 0.001	0.72(0.64–0.79)	< 0.001
Household/Community level variables						
Zone	N = 6,869					
Southern	2,929	1,171(39.6)	1.04(0.94–1.15)	0.422	1.08(0.91–1.29)	0.360
Middle (Ref.)	1,613	627(38.0)				
Northern	2,327	1,027(44.8)	1.17(1.06–1.30)	0.002	1.22(1.01–1.46)	0.031
Residence						
Urban (Ref.)	3,523	1474(42.3)				
Rural	3,481	1407(39.0)	0.92(0.85–0.99)	0.043	1.02(0.99–1.16)	0.062
Wealth quintile						
Poorest	1,678	735(44.4)	1.09(0.97–1.23)	0.110	1.36(1.01–1.83)	0.049
Poorer	1,533	646(40.6)	1.00(0.88–1.14)	0.921	1.12(1.02–1.22)	0.023
Middle (Ref.)	1,417	565(40.3)				
Richer	1,300	503(38.5)	0.97(0.83–1.08)	0.459	0.99(0.93–1.13)	0.304
Richest	1,076	432(39.4)	0.97(0.85–1.12)	0.750	0.97(0.87–1.08)	0.612
Type of toilet facility	N = 6,869					
Unimproved (Ref.)	3,152	1,386(43.4)				
Improved	3,717	1,439(38.8)	0.89(0.83–0.96)	0.003	0.90(0.83–0.96)	0.004

^6^ CPR = Crude prevalence ratio, ^7^APR = Adjusted prevalence ratio as used in the Poisson regression analysis. ^8^Ref = reference value

**Table 5:** Multivariate regression results of determinants of anaemia among pregnant women (15–49 years) in Ghana (GDHS 2022)

Background variables	pregnant women (N = 553)					
	Anaemia		Odds ratio (95% CI)			
	Number	prevalence n (%)	CPR (95% CI)	p- value	APR (95% CI)	p- value
Total	553	300(51.4)	-	-	-	-
Individual level variables						
Age						
15–19	37	23(66.9)	1.70(1.06–2.72)	0.026	1.34(0.85–2.11)	0.204
20–24	130	79(56.2)	1.43(0.94–2.17)	0.091	1.39(0.99–1.96)	0.054
25–29	150	78(57.1)	1.45(0.97–2.18)	0.069	1.26(0.93–1.69)	0.128
30–34	127	66(44.5)	1.13(0.72–1.76)	0.577	1.25(0.94–1.65)	0.112
35–39(Ref.)	86	39(39.2)				
40 +	23	15(61.2)	1.56(0.89–2.71)	0.114	1.30(0.88–1.93)	0.178
Education						
No education	135	90(69.1)	3.23(1.53–6.80)	0.002	1.19(0.66–2.15)	0.556
Primary	96	53(51.1)	2.39(1.12–5.07)	0.023	1.16(0.65–2.08)	0.601
Secondary	285	153(50.8)	2.37(1.11–5.05)	0.024	1.19(0.68–2.07)	0.538
Higher (Ref.)	37	10(21.4)				
Religion						
Christian	184	163(46.0)	0.56(0.39–0.81)	0.002	0.79(0.54–1.15)	0.221
Islamic	341	116(63.7)	0.78(0.55–1.12)	0.186	0.98(0.68–1.41)	0.916
Traditionalist	19	14(70.7)	0.87(0.58–1.29)	0.503	0.93(0.60–1.45)	0.765
Others (Ref.)	9	7(81.0)				
Number of living children						
0	119	69(56.0)	1.26(0.89–1.77)	0.180	1.15(0.88–1.49)	0.287
1 (ref.)	142	67(44.4)				
2–4	237	128(50.4)	1.13(0.84–1.53)	0.406	1.21(0.95–1.55)	0.110
5 +	55	36(68.5)	1.54(1.11–2.14)	0.009	1.43(1.02–2.00)	0.037
Body mass index (kg/m^2^)						
Underweight (< 18.5)	14	10(76.0)	1.31(0.94–1.82)	0.102	1.23(0.86–1.75)	0.246
Normal (18.5–24.9) (Ref.)	305	185(57.8)				
Overweight (25–29.9)	153	79(51.9)	0.89(0.70–1.14)	0.375	0.93(0.77–1.13)	0.487
Obese (30 +)	81	26(34.8)	0.60(0.41–0.86)	0.006	0.66(0.46–0.94)	0.025
ITN usage						
No (Ref.)	253	121(45.8)				
Yes	300	179(57.5)	1.25(1.01–1.55)	0.034	1.12(0.86–1.33)	0.246
Household/Community level variables						
Zone	N = 544					
Southern	203	99(45.7)	0.92(0.72–1.17)	0.535	1.02(0.82–1.28)	0.795
Middle (Ref.)	125	61(49.3)				
Northern	216	134(66.6)	1.35(1.09–1.67)	0.006	1.26(1.01–1.57)	0.043
Residence						
Urban (Ref.)	326	189(57.4)				
Rural	227	111(45.6)	0.79(0.65–0.96)	0.022	1.10(0.91–1.33)	0.317
Household head						
Female (Ref.)	154	73(41.0)				
Male	399	227(56.2)	1.36(1.04–1.78)	0.021	1.13(0.94–1.37)	0.182
Wealth quintile						
Poorest	161	110(72.2)	1.49(1.15–1.93)	0.002	1.32(1.01–1.76)	0.049
Poorer	128	74(55.5)	1.19(0.88–1.60)	0.240	1.14(0.88–1.48)	0.299
Middle (Ref.)	102	50(48.4)				
Richer	91	47(50.5)	0.60(0.35–1.02)	0.059	1.16(0.86–1.56)	0.310
Richest	71	19(29.2)	0.43(0.19–0.99)	0.049	0.69(0.43–1.10)	0.120
Type of toilet facility	N = 544					
Unimproved (Ref.)	276	173(64.2)				
Improved	268	121(43.5)	0.67(0.56–0.81)	< 0.001	0.94(0.77–1.15)	0.574

Nonpregnant women without living children had a 22% lower prevalence of anaemia (APR: 0.78, 95% CI 0.65–0.94, p = 0.045) compared to those with children. Among pregnant women, those with five or more living children had a 43% higher risk of anaemia (APR: 1.43, 95% CI 1.02–2.00, p = 0.037) than those without living children. The risk of developing anaemia increased with the number of living children for both groups of women.

Among nonpregnant participants, those who were underweight had an 11% higher prevalence of anaemia (APR: 1.11, 95% CI 1.01–1.22, p = 0.020), while those who were overweight and obese had 19% (APR: 0.81, 95% CI 0.75–0.88, p < 0.001) and 28% (APR: 0.72, 95% CI 0.64–0.79, p < 0.001) lower prevalence, respectively. Among pregnant women, those who were underweight had a 23% higher risk of anaemia (APR: 1.23, 95% CI 0.86–1.75, p = 0.246), although not statistically significant (p = 0.246). Conversely, pregnant women in the obese category had a significantly lower risk (34%) of anaemia (APR: 0.66, 95% CI 0.46–0.94, p = 0.025). Although underweight women in both groups were vulnerable to anaemia, the risk was higher among pregnant women.

Nonpregnant women residing in the northern zone were 22% more likely to be anaemic (APR: 1.22, 95% CI 1.01–1.46, p = 0.031) than those in the southern zone. Similarly, pregnant women in the northern zone had a 26% greater risk of anaemia (APR: 1.26, 95% CI 1.01–1.57, p = 0.043) compared to those in the southern zone.

Among nonpregnant women, those in the poorest wealth quintile had a 36% higher risk of anaemia (APR: 1.36, 95% CI: 1.01–1.83, p = 0.049) compared to those in the richest quintile (APR: 0.97, 95% CI: 0.87–1.08, p = 0.612). Likewise, pregnant women in the poorest quintile had a significantly higher prevalence of anaemia (APR: 1.32, 95% CI 1.01–1.76, p = 0.049), whereas the association for the richest quintile was not significant (APR: 0.69, 95% CI 0.43–1.10, p = 0.120).

Nonpregnant women who reported very poor health status had a 51% higher risk of anaemia (APR: 1.51, 95% CI 1.20–1.90, p < 0.001) compared to those who reported very good health status. Conversely, women who reported very good health status had a 10% lower risk (APR: 0.90, 95% CI 0.83–0.98, p = 0.022). In addition, nonpregnant women with access to improved toilet facilities had a 10% lower prevalence of anaemia (APR: 0.90, 95% CI 0.83–0.96, p = 0.004) compared to those using unimproved facilities (Tables 4**)**.

## Discussion

This study analysed secondary data from the 2022 GDHS to compare anaemia prevalence and associated risk factors among nonpregnant and pregnant WRA in Ghana. The overall anaemia prevalence among all WRA was 41.1%, with individual prevalence of 40.4% among nonpregnant women and 51.4% among pregnant women. Anaemia prevalence among WRA declined slightly from 45% in 2003 to 41.1% in 2022, whereas anaemia in pregnancy increased from 44% in 2014 to 51.4% in 2022, following a peak of 70% in 2008 in Ghana [27]. The 41.1% anaemia prevalence among WRA in our study is higher than estimates reported in an earlier study [34], as well as global averages from the World Bank (37.0%) and WHO (35.4%) [35]. Similarly, the prevalence of anaemia in pregnancy in our study exceeded several previous estimates [36–38], although it remained lower than the 73.1% reported in one study [39]. Ghana’s anaemia burden among pregnant women remains substantially lower than in countries such as Guinea and Mali, but higher than the global average [4, 40–42]. The elevated prevalence in pregnancy is particularly concerning due to the dual risks it poses to both maternal and child health. Anaemia in pregnancy may be attributed to physiological changes that increase iron requirements, compounded by factors such as poor dietary iron absorption and limited access to iron supplementation [43–45].

In this study, common determinants of anaemia among both nonpregnant and pregnant women included parity, BMI, geographic zone, and wealth quintile, while self-reported health status and type of toilet facility were specific to nonpregnant women.

The number of living children per woman (parity) is correlated with both the severity and prevalence of anaemia in nonpregnant and pregnant women. This result is consistent with evidence from multiple studies conducted in Africa and Asia [46–49]. Multiple pregnancies can deplete essential nutrients such as vitamin B12, iron, and folate that are critical for Hb synthesis [43–45]. Additionally, blood loss during childbirth increases the risk of anaemia. Ghana’s high unmet need for family planning may contribute to its elevated fertility rate (3.9 births per woman) [27, 48–50], which, in turn, can lead to adverse maternal health outcomes, including increased anaemia prevalence.

Concerning BMI, our findings align with previous studies [29, 51–53] showing that maintaining a normal weight or being overweight/obese is associated with a reduced risk of anaemia, while being underweight increases the risk among WRA. Although underweight women in both groups were predisposed to anaemia, the risk was notably higher among pregnant women. This may be attributed to undernutrition; in our study, only 49.8% of women met the minimum dietary diversity threshold. In Ghana, 23.1% of WRA live below the poverty line, and when combined with food insecurity, this could limit access to essential nutrients and a balanced diet [48–50, 54, 55].

Regarding geographic zone, our findings revealed notable regional differences in the prevalence and severity of anaemia among both nonpregnant and pregnant women. Overall, anaemia prevalence was higher in the northern zone compared to the southern zone. Women residing in the northern zone were more likely to be anaemic. This regional disparity may be attributed to lower socioeconomic status, limited access to healthcare services, and reduced affordability of nutritious diets among women in the northern zone [11, 29, 56]. In addition, nonpregnant women in Northern Ghana often engage in strenuous physical labour with limited rest, increasing energy expenditure and potentially contributing to iron loss [13, 57]. This association is consistent with earlier studies reporting similar regional disparities in anaemia prevalence [31, 58–60].

Household wealth status was associated with risk of anaemia in both nonpregnant and pregnant women. However, the risk of anaemia was higher among nonpregnant women. Compared to those in the poorest quintile, women in the richest quintile had the lowest risk of anaemia in both groups. These findings suggest that higher wealth may play a protective role against anaemia, whereas poverty appears to be associated with increased risk [17, 31, 61, 62]. This association may underscore the importance of designing targeted interventions for women in lower wealth quintiles.

Nonpregnant women who perceived their health status as poor had a significantly higher risk of anaemia compared to those who reported good health. This aligns with findings from previous studies, which have shown that self-reported poor health is often associated with limited access to healthcare services, chronic conditions such as parasitic infections, and nutritional deficiencies all of which are recognized contributors to anaemia [17, 51, 53, 63–67]. Women with poor self-rated health may thus experience a mixture of vulnerabilities that amplify their risk of anaemia, particularly in settings with constrained healthcare resources. This finding could offer a window of opportunity for public health interventions, such as raising awareness and integrating anaemia screening into routine primary healthcare services for WRA, particularly those reporting poor health status.

Nonpregnant women with improved toilet facilities had a decreased risk of anaemia compared to those with unimproved facilities. Our findings align with previous studies suggesting that improved sanitation plays a crucial role in reducing anaemia risk by lowering the incidence of infectious diseases [68, 69]. Unimproved sanitation is associated with higher risks of infections such as schistosomiasis and soil-transmitted helminthiasis, which can cause recurrent blood loss and iron depletion [70]. Additionally, poor sanitation often leads to increased exposure to diarrhoeal diseases, which can impair nutrient absorption, including that of iron [30]. This association suggests that integrating WASH interventions into anaemia prevention programmes may be beneficial, particularly in resource-limited settings.

Maternal age, though not statistically significant in the final model, appeared to influence anaemia prevalence among WRA. Our data showed higher anaemia prevalence in pregnant women across all age groups compared to nonpregnant women. Anaemia was particularly more common among younger women (15–24 years) and older women (40–49 years) in both groups. This finding corresponds with earlier research [11, 29, 71–73], which show that younger women may be at greater risk due to factors such as underweight status, socioeconomic disadvantage, unplanned pregnancies, and inadequate nutrition. Contrary, older women may face anaemia risks from malabsorption, nutritional deficiencies, and chronic health conditions. The increasing incidence of anaemia among younger females is concerning, especially given the rising rate of teenage pregnancies in Ghana. More teenage pregnancies could exacerbate anaemia prevalence and contribute to adverse outcomes, including low birth weight, preterm births, stillbirths, congenital malformations, abortions, and increased maternal and neonatal mortality [64, 65, 74]. This association suggests the need to prioritize anaemia screening and treatment, particularly among younger women.

### Strengths and limitations

This study used nationally representative data with standardized procedures, thereby enhancing the validity and accuracy of the findings. The large sample size also supports generalizing the results to the broader populations. Again, the study covered nonpregnant and pregnant women and has therefore explored the variation of anaemia determinants in the entirety of WRA. The study had some limitations as it relied on self-reported pregnancy status without laboratory confirmation, which may have introduced bias, as some women in early pregnancy might have been misclassified. The outcome variable (anaemia status) was dichotomized into ‘anaemic’ and ‘nonanaemic’ to facilitate analysis using a binary Poisson regression model. However, this simplification may have led to the loss of important information on anaemia severity (e.g. mild, moderate, severe), potentially limiting the sensitivity and statistical power to identify risk factors specifically associated with moderate or severe anaemia. Furthermore, the cross-sectional design limits the ability to establish causal relationships between exposure and outcome variables.

## Conclusion

This study revealed a higher prevalence of anaemia among nonpregnant (40.4%) and pregnant (51.4%) WRA, thereby rating it as a severe public health issue in Ghana. We identified several shared and unique determinants of anaemia among nonpregnant and pregnant women. Common risk factors that increase anaemia prevalence in both groups include high parity, low BMI, lower wealth status, and residence in the northern zone. Multiparous and underweight women, particularly those who were pregnant, were at higher risk of anaemia. Women in the richest wealth quintile were protected, while those in the poorest quintile were more vulnerable to anaemia. Specifically, nonpregnant women with good health status and access to improved toilet facilities were associated with a lower risk of anaemia. The finding suggests prioritizing interventions that enhance access to healthcare, nutrition, sanitation, and economic equity, particularly for pregnant women. Additionally, these findings underscore the importance of comprehensive contraceptive education and availability to enable women to make informed reproductive choices and effectively space their pregnancies.

## Supplementary Information

Supplementary file 1.

## Data Availability

The dataset used for the study can be uploaded from this link [75]. Further datasets used in the analysis will be made available upon request from the corresponding author.

## Abbreviations

APR: Adjusted prevalence ratio
BMI: Body mass index
CI: Confidence interval
CPR: Crude prevalence ratio
DHS: Demographic and health survey
GDHS: Ghana demographic and health survey
GNHREC: Ghana national health research ethics committee
GIFTs: Girls’ Iron-folate tablet supplementation
GSS: Ghana statistical service
Hb: Haemoglobin
ITN: Insecticide-treated net
LMICs: Low–middle-income countries
NHIS: National health insurance scheme
SD: Standard deviation
USAID: United States agency for international development
WASH: Water sanitation and hygiene
WHO: World Health Organization
WRA: Women of reproductive age
