# Supplementary material for: Anaemia prevalence and risk factors among nonpregnant and pregnant women of reproductive age in Ghana: an analysis of the Ghana demographic and health survey data
Source: Trop Med Health. 2025 Aug 27;53:118. doi: 10.1186/s41182-025-00792-8 (PMC12382169; doi:10.1186/s41182-025-00792-8)
Supplement: Supplementary file 1 [file 41182_2025_792_MOESM1_ESM.docx]

**Anaemia prevalence and risk factors among nonpregnant and pregnant women of reproductive age in Ghana: An analysis of the Ghana Demographic and Health Survey Data**

**Agulu Gilbert Gangtaba**, **Noudéhouénou** **Crédo** **Adelphe Ahissou, Yasuhiko Kamiya, Frank Baiden, Mitsuaki Matsui**

# **Supplementary Material File**

**Supplementary Table S1.** **List of exposure variables included in the study**

| **Variable** | **Category/Code** | **Scale** | **Definition/Description** |
| --- | --- | --- | --- |
| **Household/Community level variables** | | | |
| Geographic Zone | Southern=1  Middle=2  Northern=3 | Nominal | The geographic zone women hail from during data collection. Ghana has 16 administrative regions. In this study, the regions have been categorized into three strata (Southern, Middle and Northern). |
| Place of residence | Urban=1  Rural=2 | Binary | Dwelling place of women, the night before the data collection. |
| Sex of Household Head | Female=0  Male=1 | Binary | Household head is described as a leader who makes decision in the household. |
| Partner occupational level | Not employed=0  employed=1 | Binary | The employment status of the woman’s partner |
| Wealth index | Poorest=1  Poorer=2  Middle=3  Richer=4  Richest=5 | Ordinal | A score is allotted to households based on the number of goods they possess [27, 51, 53, 67]. This was calculated using the principal components analysis. Details on wealth index information can be found in the GDHS report. |
| Source of drinking water | Unimproved=1  Improved=2 | Binary | A household was said to have an improved drinking water source when it had: piped water, public taps, standpipes, tube wells, boreholes, protected dug wells and springs, rainwater, water delivered via tanker, bottled, and sachet water [27, 51, 53, 67]. |
| Sanitation facility | Unimproved=1  Improved=2 | Binary | An improved sanitation facility was said to be achieved when a household possesses; flush/pour toilets that flush water and waste to a piped sewer system, septic tank, pit latrine, or unknown destination, ventilated improves, latrines, pit latrines with slabs, and composting toilets [27, 51, 53, 67]. |
| **Individual level Variables** | | | |
| Age (years) | 15-19=1  20-24=2  25-29=3  30-34=4  35-39=5  40-44=6  45-49=7 | Nominal | Age in completed years of women during the data collection period. |
| Marital status | No=0  Yes=1 | Binary | This variable defines whether a woman was legally married or cohabitating with a partner before the survey. |
| Birth experience | ≤24 months=1  ≥25 months=2 | Binary | The interval between the last birth experienced by women and the start of the DHS data collection |
| Pregnancy Status | Not pregnant=0  Pregnant=1 | Binary | The operational definition of pregnancy in this study is between a period of a confirmed pregnancy test to 40 weeks of gestation. |
| Dietary Diversity | No=0  Yes=1 | Binary | This indicator was calculated based on ten food groups: grains, white/pale starchy roots, tubers, and plantains; pulses (beans, peas, and lentils); nuts and seeds; dairy (milk and milk products); flesh foods (meat, fish, poultry, and organ meat); eggs; dark green leafy vegetables; vitamin-A rich fruits and vegetables; other vegetables; and other fruits. Women who consumed at least five/10 food groups in the 24 hours before the survey were classified as having minimally adequate dietary diversity [27, 51, 53, 67]. |
| Education level | No education=0  Primary=1  Secondary=2  Higher=3 | Ordinal | The level of educational attainment during the survey. In this study, education has been categorized into primary, secondary, higher, or no education. |
| Religion | Islamic=0  Christianity=1  Traditionalist=2  Others=3 | Binary | This variable defines the religion women were affiliated to during the data collection. |
| Type of employment | Unemployed=0  Government employee=1  Self-employed=2 | Nominal | "Type of employment" pertains to the women’s current work situation and whether employed full-time, on contract, part-time, self-employed, governmental, or unemployed. |
| Parity | Nulliparity=0  Primiparity=1  Multiparity=2  Grand-multiparity=3 | Ordinal | This variable defines the childbirth experienced by women. Nulliparity refers to those without any childbirth experience. Primiparity refers to those with one child. Multiparity are women who have less than 5 children and grand-multiparity are those with five or more children |
| Self-reported Health Status | Very bad=1  Bad=2  Moderate=3  Good=4  Very good=5 | Ordinal | This indicator explains the health state of the women during data collection as verbalized by the women. |
| Literacy rate | Yes=1  No=0 | Binary | Literacy rate is defined in this study as the ability of participants to read a sentence. |
| Alcohol use | No=0  Yes=1 | Binary | The use of any form of alcoholic drink, beverage, or liquor at least within one month preceding the 2022 GDHS data collection. |
| Body Mass Index (BMI) | Underweight=1  Normal=2  Overweight=3  Obese=4 | Ordinal | BMI was defined as the ratio of weight relative to height squared. With categorizations as:  Underweight (BMI<18.5); Normal (BMI=18.5-24.9); Overweight(BMI=25.0-29.9) Obese (BMI≥30) [27, 51, 53, 67]. |
| Usage of ITN | No=0  Yes=1 | Binary | This defines the utilization rate of an Insecticide Treated Net (ITN) during the survey. |
| Health Insurance (HI) coverage | No=0  Yes=1 | Binary | A “yes” response defines women with a valid HI card during the survey. Those without a HI card or lost or expired card were categorized as “No” [27, 51, 53, 67]. |

**Supplementary Table S2. Results of variance inflation factor (VIF)**

#

| Variable | VIF | 1/VIF |
| --- | --- | --- |
| Age |  |  |
| 1 | 2.38 | 0.420292 |
| 2 | 1.87 | 0.535879 |
| 3 | 1.65 | 0.606994 |
| 4 | 1.47 | 0.681605 |
| Parity |  |  |
| 0 | 1.35 | 0.740455 |
| 1 | 2.39 | 0.418220 |
| 2 | 2.94 | 0.340617 |
| Health status |  |  |
| 1 | 1.96 | 0.510130 |
| 2 | 1.98 | 0.505683 |
| 3 | 1.17 | 0.855017 |
| 4 | 1.04 | 0.965926 |
| ITN use | 1.16 | 0.863020 |
| Toilet Facility | 1.67 | 0.597080 |
| Education |  |  |
| 0 | 7.20 | 0.138902 |
| 1 | 4.35 | 0.230010 |
| 2 | 5.14 | 0.194594 |
| BMI |  |  |
| 1 | 1.05 | 0.948121 |
| 2 | 1.26 | 0.795646 |
| 3 | 1.40 | 0.711884 |
| Geographic Zone |  |  |
| 1 | 1.97 | 0.506795 |
| 2 | 1.93 | 0.519250 |
| Residence | 1.67 | 0.597854 |
| Household Head | 1.05 | 0.949702 |
| Wealth quintile |  |  |
| 1 | 1.62 | 0.616805 |
| 2 | 2.20 | 0.453905 |
| 3 | 2.69 | 0.372174 |
| 4 | 3.34 | 0.299390 |
| Partner occupation | 1.03 | 0.970680 |
| Alcohol consumption | 1.03 | 0.970398 |
| NHIS use | 1.05 | 0.953696 |
| Woman occupation |  |  |
| 0 | 1.12 | 0.891511 |
| 1 | 1.20 | 0.836799 |
| Literacy | 2.42 | 0.413148 |
| Water source | 1.26 | 0.795683 |
| MDD | 1.06 | 0.943296 |
| Mean VIF | 2.00 |  |
